# Urine concentration impairment in sickle cell anemia: genuine nephrogenic diabetes insipidus or osmotic diuresis?

**DOI:** 10.1152/ajprenal.00313.2023

**Published:** 2023-12-07

**Authors:** Quentin de Berny, Camille Saint-Jacques, Aline Santin, Sarah Mattioni, Olivier Steichen, Rémi Chieze, Vincent Frochot, Emmanuel Letavernier, François Lionnet, Jean-Philippe Haymann

**Affiliations:** ^1^Service de Néphrologie, Dialyse et Transplantation Rénale, Centre Hospitalier Universitaire d’Amiens, Amiens, France; ^2^Service d'Explorations Fonctionnelles Multidisciplinaires, Hôpital Tenon, Assistance Publique-Hôpitaux de Paris, UMR-S 1155, Médecine Sorbonne Université, Paris, France; ^3^Service de Médecine Interne, Hôpital Tenon, Assistance Publique Hôpitaux de Paris, Médecine Sorbonne Université, Paris, France

**Keywords:** antidiuretic hormone, hyposthenuria, nephrogenic diabetes insipidus, sickle cell anemia, urine concentration impairment

## Abstract

The urine concentration impairment responsible for hyposthenuria in sickle cell nephropathy is currently thought to be a consequence of renal medulla lesions, which lead to nephrogenic diabetes insipidus. The objective of the present study was to investigate the mechanism of hyposthenuria in patients with sickle cell anemia. We performed an observational study of patients with homozygous SS sickle cell anemia and data available on the fasting plasma antidiuretic hormone (ADH) concentration. A total of 55 patients were analyzed. The fasting plasma ADH values ranged from 1.2 to 15.4 pg/mL, and 82% of the patients had elevated ADH values and low fasting urine osmolality (<505 mosmol/kgH_2_O). Plasma ADH was positively associated with plasma tonicity and natremia (*P* < 0.001). None of the patients experienced polyuria and fasting free water clearance was negative in all cases, thus, ruling out nephrogenic diabetes insipidus. The tertile groups did not differ with regard to fasting urine osmolality, plasma renin level, mGFR, or several hemolysis biomarkers. The negative fasting free water clearance in all cases and the strong association between 24-h osmolal clearance and 24-h diuresis favors the diagnosis of osmotic diuresis due to an impaired medullary gradient, rather than lesions to collecting tubule.

**NEW & NOTEWORTHY** The urine concentration impairment in sickle cell anemia is an osmotic diuresis related to an impaired renal medullary gradient leading to an ADH plateau effect. The fasting plasma ADH was high in the context of a basic state of close-to-maximal urine concentration probably driven by short nephrons maintaining a cortex-outer medullary gradient (about 400 milliosmoles). The patients had a low daily osmoles intake without evidence of thirst dysregulation so no one experienced polyuria.

## Graphical abstract

**Figure:**
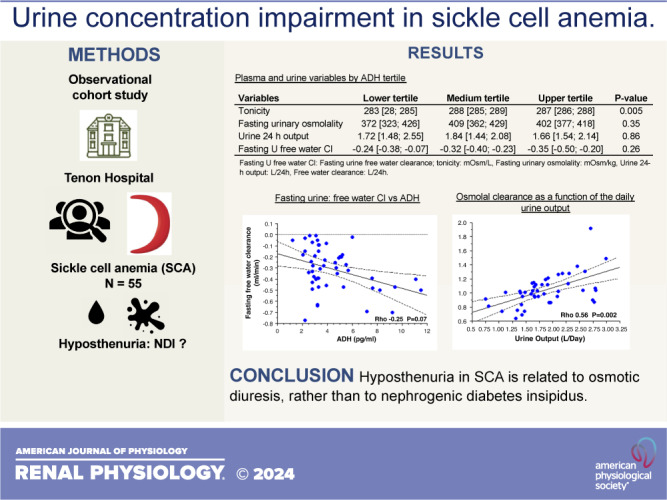

## INTRODUCTION

Sickle cell anemia (SCA) is a genetic disorder defined by the presence of a mutation in the *β-globin* gene of hemoglobin (HbS). The disease is common, especially in African regions (1), but has spread worldwide as people migrate (2). SCA is a systemic disease (3). HbS polymerization in red blood cells generates chronic hemolytic anemia and acute vaso-occlusion, which both lead to organ damage. Sickle cell nephropathy includes glomerular damage—characterized by hyperfiltration, albuminuria—tubular injury (4, 5), and, ultimately, kidney failure. Inner medulla lesions (6) appear to be a hallmark of sickle cell nephropathy, as local hypoxia and acidosis promote HbS polymerization in the vasa recta. Hence, vasa recta rarefaction may account for medullary gradient loss, a high prevalence of renal tubular acidosis, decreased NH_4_ excretion, and a urine concentration impairment (UCI) referred to as hyposthenuria. The latter disorder was first reported in SCA in 1928 and can occur from early childhood onward (7). In addition to vasa recta rarefaction, tubular lesions may also occur and can lead to metabolic acidosis and polyuria. Although SCA is listed as a cause of nephrogenic diabetes insipidus (NDI), data are lacking, hence the mechanism of hyposthenuria needs to be clarified. In the present study, we assessed the prevalence of polyuria, and measured urine concentrating parameters and the fasting plasma antidiuretic hormone (ADH) to unravel the mechanism of the UCI in patients with SCA.

## MATERIALS AND METHODS

### Patients and Study Design

**Figure 1.**
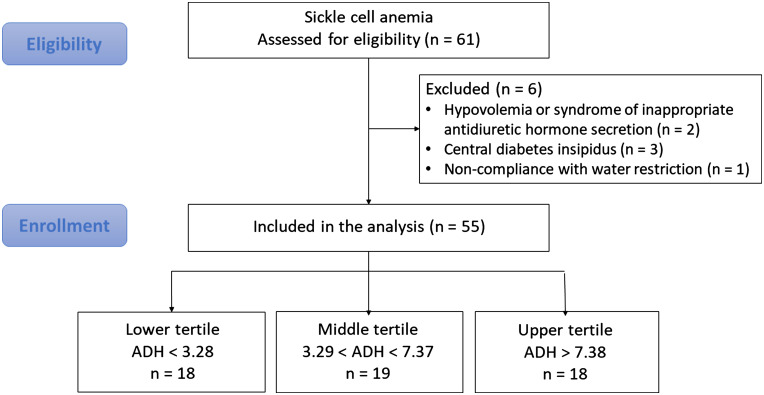
Flow chart. The ADH level is expressed in pg/mL. ADH, antidiuretic hormone. *n*, number of patients.

This observational study included 61 patients with homozygous SS SCA referred for a routine renal and metabolic workup in our department of renal physiology (Hôpital Tenon, Assistance Publique Hôpitaux de Paris, Paris, France). All the patients received regular medical care at the hospital’s sickle cell center. The routine evaluations included measurement of the glomerular filtration rate (mGFR), a clinical examination, laboratory tests (including the fasting plasma ADH concentration), and fasting and 24-h urine collection. Six patients (9.8%) were excluded: two patients had fasting hyponatremia (natremia < 138 mEq/L) and a high plasma ADH value (>4 pg/mL) that suggested the presence of hypovolemia or syndrome of inappropriate antidiuretic hormone secretion; three patients with hypernatremia (natremia ≥ 144 mEq/L) and low ADH (ADH < 2.5 pg/mL) suggesting central diabetes insipidus and one patient who had clearly not fasted (the fasting urine osmolarity was lower than the 24-h urine osmolarity). Thus, 55 patients were included in the final analysis (Fig. 1). All participants gave their written, informed consent. In line with the French legislation on observational studies of routine clinical practice, approval by an institutional review board was not required. However, the study data were registered and approved by the French National Data Protection Commission (Commission nationale de l’informatique et des libertés, Paris, France; reference: 2065902v0).

### Data Collection and Measurements

As mentioned above in *Patients and Study Design*, 5-h in-person visits provided a complete nephrological work-up (8), including the mGFR determined from renal clearance of ^51^Cr-EDTA (9) and standardized against the body surface area. On admission, patients had fasted for at least 8 h. Laboratory tests were run in our unit’s laboratory or the hospital’s central laboratory. The clinical variables included age, sex, body mass index (BMI), blood pressure, and heart rate. The laboratory test variables included a fasting blood sample, a fresh urine sample, and 24-h urine collection. The urine concentration ability was tested using fasting urine osmolality. The renal handling of water and osmoles was depicted by fasting free water clearance, fasting osmolal clearance, 24-h free water clearance, 24-h osmolar clearance, and 24-h urine output. The hormonal regulation of urine concentrating was determined by plasma osmolarity, natremia with regard to fasting plasma ADH. Furthermore, we used 24-h urine osmoles to determine the daily osmole intake and 24-h urine urea excretion for the daily protein intake. Plasma and urine osmolalities were measured using an Osmometer 3320 (Advanced Instruments Inc., Norwood, MA). Plasma tonicity (mosmol/kgH_2_O) was calculated as twice the natremia plus the fasting blood glucose. Osmolal clearance was calculated as 24 h-urine osmolality (mosmol/day) divided by the plasma tonicity (mosmol/kgH_2_O). Free water clearance was calculated as the difference between the 24-h urine output (L/day) and osmolal clearance (L/day). Osmolal and free water clearances were converted into mL/min. Plasma ADH levels were determined in duplicate in a radioimmunoassay, as reported previously (10). Sodium, potassium, chloride, ionized calcium, blood and urine pH, and bicarbonate concentrations were measured with a pH/blood-gas analyzer (ABL 800, FLEX, Radiometer, Copenhagen, Denmark). Plasma levels of urea, creatinine, and proteins at baseline were measured with routine laboratory techniques. Plasma renin concentrations were determined in an enzyme immunoassay (Immunodiagnostic Systems, France).

### Statistical Analyses

All statistical analyses were performed with XLSTAT software (Addinsoft, Paris, France). Quantitative data were expressed as the median [interquartile range]. Qualitative data were expressed as the frequency (percentage). The study participants were divided into three ADH tertiles and compared using a nonparametric Kruskall–Wallis test. Correlations were evaluated using a nonparametric Spearman test. The threshold for statistical significance was set to *P* < 0.05. Figures were produced with StatView software (SAS Institute Inc., Cary, NC).

## RESULTS

### Baseline Characteristics

In the study population, the median age was 41.4, the male/female ratio was 22/33, the median BMI was 23.9 kg/m^2^, and seven (13%) of the patients were obese (BMI > 30 kg/m^2^). None of the patients had hypertension or diabetes. The median systolic and diastolic blood pressure values were 123 [112; 130] mmHg and 68 [59; 73] mmHg, respectively. The ADH tertile groups did not differ significantly in terms of demographic and clinical data (Table 1).

**Table 1.** Demographic and clinical data, overall and by ADH tertile group

Variables	Overall *n* = 55	Lower Tertile *n* = 18	Middle Tertile *n* = 19	Upper Tertile *n* = 18	*P* Value
Age, yr	41.4 [32.2; 47.3]	42.6 [24.4; 48.2]	40.9 [36.0; 46.6]	38.9 [32.8; 44.9]	0.72
Sex (%male)	22 (40.0)	6 (33.3)	7 (36.8)	9 (50.0)	0.56
Weight, kg	69.0 [63.2; 79.7]	71.5 [69.0; 76.0]	67.5 [65.0; 79.2]	66.5 [58.5; 79.7]	0.64
BMI, kg/m²	23.9 [21.7; 26.8]	23.9 [22.3; 27.3]	23.9 [22.4; 26.0]	23.2 [20.2; 27.6]	0.59
SBP, mmHg	123 [112; 130]	126 [118; 130]	124 [112; 136]	119 [109; 127]	0.23
DBP, mmHg	68 [59; 73]	67 [61; 69]	72 [64; 77]	62 [56; 70]	0.06
BP, beats/min	73 [66; 78]	72 [66; 79]	73 [65; 77]	76 [66; 79]	0.88

ADH, antidiuretic hormone; BMI, body mass index; BP, blood pulses; DBP, diastolic blood pressure; SBP, systolic blood pressure.

### Urine Concentration Ability and Renal Handling of Water and Osmoles

**Figure 2.**
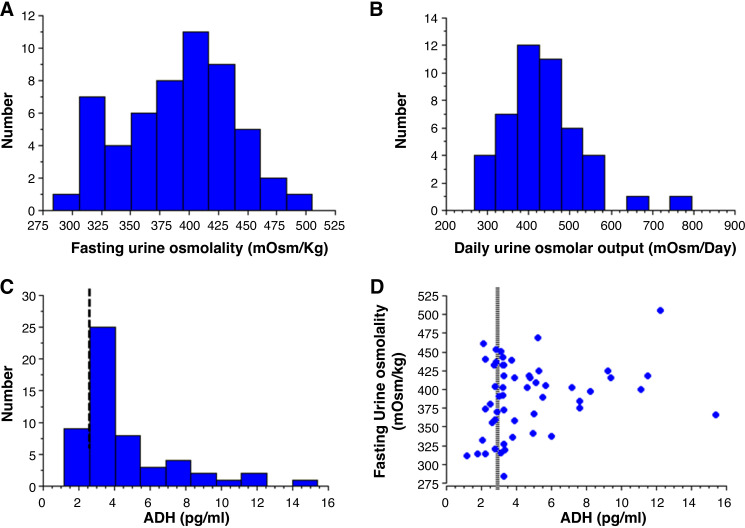
The fasting urine osmolality (*A*), daily urine osmole output (*B*), and ADH plasma concentration (*C*) in patients with SCA. The fasting urine osmolality, as a function of the plasma ADH concentration (*D*). ADH, antidiuretic hormone; SCA, sickle cell anemia.

The fasting urine osmolality was low in all cases, ranging from 284 to 505 mosmol/kgH_2_O (Fig. 2). There were no significant differences between the three groups of ADH tertiles in terms of fasting urine osmolality, free water and osmolal clearances or 24-h urine output, osmoles, and urea despite an elevated urea concentration in the upper tertile. The 24-h urine osmole concentration was also low and ranged from 223 to 404 mosmol/kgH_2_O; this meant that the difference between the 24-h urine osmole concentration and the fasting urinary osmolality was also unexpectedly low and ranged from 11 to 219 mosmol/kgH_2_O, with 80% of patients below 100 mosmol/kgH_2_O overall (but similar proportion in each tertile). None of the patients experienced polyuria, defined as a urine output above 3 L/day. The daily osmole intake was always below 600 mosmol/day, except for two patients. Accordingly, daily protein intakes were also low and ranged from 0.3 to 1.3 g/kg/day; only eight (17.4%) patients had a value above 1 g/kg/day.

**Figure 3.**
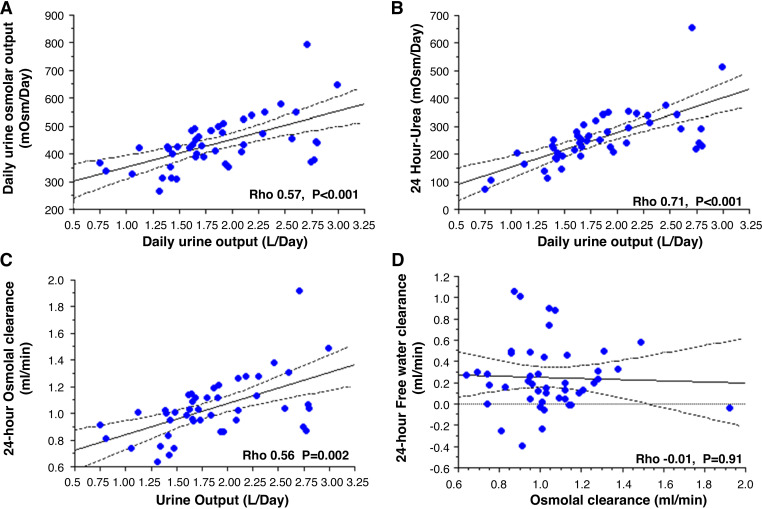
Daily urine osmolar output (*A*), 24-h urea clearance (*B*), and osmolal clearance (*C*) as a function of the daily urine output. Free water clearance as a function of the osmolal clearance (*D*). Nonparametric Spearman test was used for correlation with Rho as correlation coefficient. Statistical significance was defined as *P* < 0.05.

**Figure 4.**
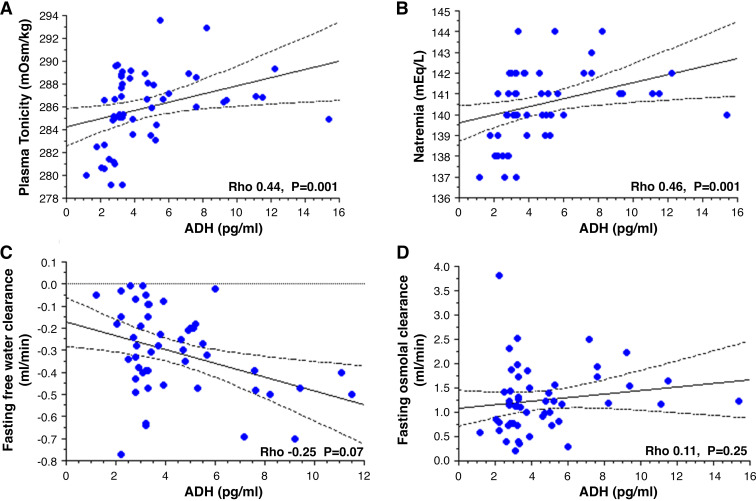
Plasma tonicity (*A*), natremia (*B*), fasting free water clearance (*C*), and fasting osmolal clearance (*C* and *D*, respectively, calculated from fasting urine collections), as a function of the ADH concentration. Nonparametric Spearman test was used for correlation with Rho as correlation coefficient. Statistical significance was defined as *P* < 0.05. ADH, antidiuretic hormone.

Twenty-four hour urine output was significantly positively associated with osmolar diet (i.e., 24-h urine osmole excretion, *P* < 0.001), daily protein intake (i.e., 24-h urine urea excretion, *P* < 0.001), 24-h urine osmolal clearance (*P* = 0.002), and also 24-h urine free water clearance (*P* = 0.002) (Fig. 3). It is noteworthy that the fasting free water clearance was always below 0 mL/min (Fig. 4*C*) and the 24-h free water clearance was always below 1.2 mL/min, with no association with 24-h osmolal clearance (Fig. 3*D*).

### Hormonal Regulation

The plasma ADH values ranged from 1.2 to 15.4 pg/ml, and 45 (82%) were considered to be high (i.e., >2.8 pg/mL). As expected, the ADH tertile groups (Table 2) showed significant differences in natremia and tonicity, with greater values in the higher tertiles. Only two (3.8%) patients had a plasma tonicity above 290 mosmol/kgH_2_O but below 295 mosmol/kgH_2_O in all cases (Fig. 4*A*). ADH was positively associated with plasma tonicity and natremia (*P* = 0.001) but not significantly correlated with fasting free water clearance (*P* = 0.07), fasting osmolal clearance (*P* = 0.25), or fasting urine osmolality (*P* = 0.16) (Fig. 4).

**Table 2.** Plasma and urine variables, overall and by ADH tertile group

Variables	Overall *n* = 55	Lower Tertile *n* = 18	Middle Tertile *n* = 19	Upper Tertile *n* = 18	*P* Value
*Plasma variables*					
ADH, pg/mL	3.30 [2.82; 5.25]	2.65 [2.20; 2.81]	3.30 [3.25; 3.85]	7.38 [5.35; 9.35]	<0.001
mGFR, mL/min/1.73 m²	82 [68; 120]	80 [48; 131]	95 [69; 120]	80 [68; 104]	0.84
Natremia, mEq/L	141 [140; 142]	139 [138; 140]	141 [140; 142]	141 [140; 142]	0.003
Tonicity, mosmol/kgH_2_O	287 [284; 289]	283 [281; 285]	288 [285; 289]	287 [286; 288]	0.005
Kalemia, mEq/L	4.2 [4.0; 4.6]	4.5 [4.0; 4.7]	4.1 [3.8; 4.3]	4.2 [4.0; 4.6]	0.10
Ionized calcium, mg/dL	4.92 [4.81; 5.05]	4.93 [4.85; 5.09]	4.81 [4.77; 4.97]	4.97 [4.93; 5.05]	0.09
Plasma CO_2_t, mEq/L	25 [24; 26]	25 [24; 26]	25 [24; 27]	25 [25; 26]	0.85
Plasma renin, pg/mL	12.0 [6.4; 31.9]	26.1 [7.5; 52.6]	7.4 [4.4; 14.2]	15.2 [10.7; 38.1]	0.05
Plasma aldosterone, pg/mL	79.0 [52.0; 147.0]	110.5 [73.2; 214.5]	81.0 [60.7; 121.7]	64.5 [46.0; 79.0]	0.10
Hemoglobin, g/dL	8.8 [8.1; 9.5]	9.2 [8.3; 9.8]	8.8 [7.8; 9.2]	8.6 [8.1; 9.3]	0.44
LDH, IU/L	403 [333; 518]	402 [339; 522]	384 [288; 505]	428 [347; 540]	0.41
Total bilirubin, µmol/L	39 [18; 57]	28 [17; 42]	35 [17; 49]	50 [26; 86]	0.13
*Urine variables*					
24 h output, L/24 h	1.73 [1.46; 2.14]	1.72 [1.48; 2.55]	1.84 [1.44; 2.08]	1.66 [1.54; 2.14]	0.86
Fasting osmolality, mosmol/kgH_2_O	399 [359; 423]	372 [323; 426]	409 [362; 429]	402 [377; 418]	0.35
Fasting U - Osm Cl, mL/min	1.18 [0.80; 1.59]	1.15 [0.72; 1.42]	1.19 [0.99; 1.54]	1.29 [0.89; 1.70]	0.61
Fasting U - Free water Cl, mL/min	−0.31 [−0.47; −0.18]	−0.24 [−0.38; −0.07]	−0.32 [−0.40; −0.23]	−0.35 [−0.50; −0.20]	0.26
24 h urine osmoles, mosmol/24 h	426 [381; 482]	390 [359; 433]	427 [400; 475]	446 [420; 513]	0.24
24 h U - Osm Cl, L/24 h	1.47 [1.31; 1.62]	1.37 [1.24; 1.49]	1.46 [1.37; 1.61]	1.54 [1.43; 1.76]	0.27
24 h U - Free water Cl, L/24 h	0.29 [0.07; 0.63]	0.37 [0.22; 0.72]	0.29 [0.07; 0.69]	0.19 [0.07; 0.37]	0.49
24 h osmolality, mosmol/kgH_2_O	333 [283; 355]	303 [257; 334]	338 [284; 363]	342 [323; 354]	0.14
Δfasting U−24 h U, mosmol/kgH_2_O	67 [40; 91]	86 [61; 99]	68 [42; 91]	47 [27; 68]	0.07
Sodium, mEq/24 h	112 [96; 143]	100 [89; 132]	116 [103; 148]	112 [96; 142]	0.26
Potassium, mEq/24 h	39 [31; 46]	43 [30; 50]	37 [21; 40]	39 [36; 48]	0.12
Urea, mmol/L	141 [116; 162]	136 [109; 158]	132 [113 ; 146]	164 [137; 176]	0.03
Urea, mmol/24 h	245 [206; 304]	232 [198; 290]	239 [194; 280]	280 [228; 331]	0.24
Uric acid, mmol/L	1.68 [1.42; 2.27]	1.54 [1.25; 1.98]	1.72 [1.29; 2.40]	1.90 [1.54; 2.34]	0.12
Fasting NH_4_^+^, mmol/L	9.94 [7.27; 17.0]	9.30 [4.9; 15.7]	9.19 [5.99; 17.7]	12.2 [8.4; 15.8]	0.77
Fasting pH	5.65 [5.33; 6.27]	5.55 [5.26; 6.23]	6.11 [5.45; 6.57]	5.60 [5.33; 6.04]	0.24
ACR, mg/mmol	4.06 [1.50; 9.91]	2.56 [1.39; 8.36]	4.95 [1.96; 15.67]	2.13 [1.37; 9.54]	0.61

ACR, albuminuria-to-creatinine ratio; ADH, antidiuretic hormone; Fasting U, fasting urine; free water Cl, free water clearance; mGFR, measured glomerular filtration rate; osm Cl, osmolal clearance; 24 h U, 24-hour urine.

### Others Biological Variables

Statistically significant intergroup differences were not found for plasma renin, mGFR, hemoglobin, and hemolytic biomarkers (plasma bilirubin and LDH).

## DISCUSSION

Even though 89% of the patients had a fasting urinary osmolality below 450 mosmol/kgH_2_O [i.e., far below the normative values of 805–867 mosmol/kgH_2_O (11) in a healthy population], the plasma ADH value was high in most of our patients with SCA. Indeed, our ADH internal control standard is a pooled sample from fasting blood donors, so the upper value of 2.8 pg/mL for the ADH concentration may be considered the highest normal fasting value [99% confidence interval]. Values of the same magnitude have been found in healthy populations, ranging from 0.87 pg/mL to a maximum of 5.42 pg/mL (albeit after 20 h of water deprivation in the latter case) (12). In our study, the ADH values in the lower tertile can be considered normal. The fasting plasma ADH concentration was above 3.0 pg/mL in 69.1% of our study participants and above 5.0 pg/mL in 29.1%. To the best of our knowledge, there are no publications on plasma ADH concentration in patients with SCA. However, some researchers have noted that plasma level of copeptin [i.e., a stable peptide derived from the precursor of vasopressin (13)] was higher in patients with SCA and a high comorbidity burden (14) but did not analyze the relationship with hyposthenuria. The 24-h urine osmole concentration in our SCA population (median [IQR]: 333 [283; 355] mosmol/kgH_2_O) was lower than in a healthy population (mean ± standard deviation: 631 ± 221 mosmol/kgH_2_O) (15) and was very close to the fasting urinary osmolality; this reflects a basic state of close-to-maximal urine concentration round the clock in patients with SCA and thus little room for further concentration. ADH osmoregulation appeared to be normal; as expected, the fasting plasma ADH concentration was positively associated with plasma tonicity and natremia. Conversely, the lack of association between fasting plasma ADH and fasting urine osmolality or free water clearance suggests the presence of kidney resistance to ADH’s action or a plateau effect. Indeed, the fasting urine osmolality and free water clearance were similar in the ADH tertile groups and were close to the osmolality value in the renal medulla. This finding suggests a plateau effect, rather than ADH inefficiency; in the latter case, one would expect to see positive free water clearance (i.e., a urine osmolality below that of the plasma) in most patients. In fact, a plateau was previously reported in patients with SCA because the administration of vasopressin did not significantly increase their urine concentration ability (7). As suggested by van Eps et al. (6), this plateau might be due to the maintenance of a cortex-outer medullary gradient by short nephrons (giving a maximum medulla osmolality of 400 milliosmoles). In contrast, long nephron function would be impaired by the vasa recta rarefaction caused by vaso-occlusive events and chronic hemolysis. Our data in humans are in line with a report on a murine model of SCA, in which an elevated vasopressin concentration in the basal state was associated with a modest increase in urine concentration after water restriction (16). In contrast to the situation in NDI, levels of aquaporin 2 (AQP2), urea transporter A1, Na-K-2Cl cotransporter (NKCC2), and epithelial Na channel were elevated in the SCA mice’s kidneys. Effectively, urine concentration requires water to shift from tubular lumen to peritubular capillaries via AQP2, 3, and 4 in collecting ducts, driven by osmotic gradient obtained by accumulation of urea and sodium in the renal medulla. Although the onset of osmotic gradient is driven by NKCC2 activation in medullary thick ascending limb of Henle’s loop, its maintenance depends upon a counter-current phenomenon requiring undamaged vasa recta (17, 18). Accordingly, we speculate that a high plasma ADH may account for a preserved or increased NKCC2 activity as previously reported (19, 20), aiming to enhance cortex-papillary gradient as much as possible. Indeed, the renal medulla is reported to be damaged, with vasa recta rarefaction (especially in the inner medulla and renal papilla) with potential additional lesions within collecting tubules. Despite the presence of an impaired urine concentration ability [in line with previous reports that referred to this disorder as hyposthenuria (21–23)], none of the patients in our series experienced polyuria. However, 17.4% of the patients had a urine output above 2.5 L/day. This apparent discrepancy might be explained by *1*) a moderate impairment of urine concentration in the context of a low osmolar diet and *2*) normal thirst regulation. Indeed, the median osmolar diet value was 426 mosmol/day; hence, a fasting urine osmolality within the 300–400 mosmol/kg range would, in theory, require between 1 and 2 L of urine. Although we did not score the patients’ thirst in the fasting state, the fact that the fasting and 24-h urine osmolar concentrations were very similar (Table 2) suggests that water intake was adjusted to match the plasma tonicity and that there was no obvious thirst reset in our study population. With a higher osmolar diet, the increase in osmolal clearance was strongly and positively associated with 24-h diuresis but was not linked to the 24-h free water clearance value, which was below 1.2 mL/min in all cases (median: 0.2 mL/min). Thus, water intake was indeed adjusted to the osmolar intake to avoid thirst and, ultimately, maintain normal plasma tonicity. This situation differs from typical NDI, in which positive free water clearance is due to low AQP2 membrane expression in the collecting tubules (24).

### Study Limitations

Unfortunately, routine renal imaging to assess papillary necrosis and daily water intake was not available in our patients, and fasting thirst was not scored. However, the participants’ thirst threshold was probably normal (which would rule out polydipsia or adipsia) because *1*) most patients had a normal fasting plasma tonicity and no polyuria and *2*) the fasting and 24-h urine osmolar concentrations were very similar (less than 50 mosmol/kgH_2_O) in all groups. Hypovolemia could be reasonably ruled out as a potential confounding factor accounting for ADH concentration increase because the plasma renin and aldosterone levels were not increased in the upper tertile group. Although we suspect that osmoreceptor sensitization was not present in the upper tertile, we cannot rule it out; specific investigations would be required. In our adult patients with SCA, the ADH level was not associated with anemia or levels of hemolysis biomarkers; this contrasts with studies of children with SCA in whom sequential blood transfusions or treatment with hydroxyurea were able to partially reverse the UCI (7, 25). However, a UCI in young patients can be reversed by an increase in renal hemodynamics (including those in the vasa recta) following the correction of anemia and a reduction in chronic hemolysis. Interestingly, Francis et al. (26) reported a fast decline of urine osmolality with increasing age of patients with sickle cell and with a lesser degree in those with sickle trait. Lastly, and despite the absence of an association between the plasma ADH concentration and the mGFR, our observational data do not tell us whether the elevated plasma ADH observed in our patients with SCA might be a risk factor for CKD progression, as shown in others kidney diseases (27).

In conclusion, our data showed that most adult patients with SCA had a mild UCI but none had a positive fasting free water clearance. In most cases, our findings were suggestive of an impairment in the corticomedullary gradient, rather than genuine NDI. The elevated fasting plasma ADH concentration in most patients with SCA thus appears to be related to osmotic diuresis rather than true NDI, with no evidence of thirst center dysregulation.

## DATA AVAILABILITY

The data that support the findings of this study are available from the corresponding author J-P.H., upon reasonable request.

## DISCLOSURES

No conflicts of interest, financial or otherwise, are declared by the authors.

## AUTHOR CONTRIBUTIONS

Q.d.B., F.L., and J-P.H. conceived and designed research; Q.d.B. and J-P.H. analyzed data; Q.d.B. and J-P.H. prepared figures; Q.d.B. and J-P.H. drafted manuscript; Q.d.B., C.S-J., A.S., S.M., O.S., R.C., V.F., E.L., F.L., and J-P.H. edited and revised manuscript; Q.d.B., C.S-J., A.S., S.M., O.S., R.C., V.F., E.L., F.L., and J-P.H. approved final version of manuscript.
